# *Legionella pneumophila *infection induces programmed cell death, caspase activation, and release of high-mobility group box 1 protein in A549 alveolar epithelial cells: inhibition by methyl prednisolone

**DOI:** 10.1186/1465-9921-9-39

**Published:** 2008-05-01

**Authors:** Makoto Furugen, Futoshi Higa, Kenji Hibiya, Hiromitsu Teruya, Morikazu Akamine, Shusaku Haranaga, Satomi Yara, Michio Koide, Masao Tateyama, Naoki Mori, Jiro Fujita

**Affiliations:** 1 Department of Medicine and Therapeutics, Control and Prevention of Infectious Diseases, Graduate School of Medicine, University of the Ryukyus, 207 Uehara, Nishihara-Town, Okinawa 903-0215, Japan; 2 Department of Molecular Virology and Oncology, Graduate School of Medicine, University of the Ryukyus, 207 Uehara, Nishihara-Town, Okinawa 903-0215, Japan

## Abstract

**Background:**

*Legionella pneumophila *pneumonia often exacerbates acute lung injury (ALI) and acute respiratory distress syndrome (ARDS). Apoptosis of alveolar epithelial cells is considered to play an important role in the pathogenesis of ALI and ARDS. In this study, we investigated the precise mechanism by which A549 alveolar epithelial cells induced by *L. pneumophila *undergo apoptosis. We also studied the effect of methyl prednisolone on apoptosis in these cells.

**Methods:**

Nuclear deoxyribonucleic acid (DNA) fragmentation and caspase activation in *L. pneumophila*-infected A549 alveolar epithelial cells were assessed using the terminal deoxyribonucleotidyl transferase-mediated triphosphate (dUTP)-biotin nick end labeling method (TUNEL method) and colorimetric caspase activity assays. The virulent *L. pneumophila *strain AA100jm and the avirulent *dotO *mutant were used and compared in this study. In addition, we investigated whether methyl prednisolone has any influence on nuclear DNA fragmentation and caspase activation in A549 alveolar epithelial cells infected with *L. pneumophila*.

**Results:**

The virulent strain of *L. pneumophila *grew within A549 alveolar epithelial cells and induced subsequent cell death in a dose-dependent manner. The avirulent strain *dotO *mutant showed no such effect. The virulent strains of *L. pneumophila *induced DNA fragmentation (shown by TUNEL staining) and activation of caspases 3, 8, 9, and 1 in A549 cells, while the avirulent strain did not. High-mobility group box 1 (HMGB1) protein was released from A549 cells infected with virulent *Legionella*. Methyl prednisolone (53.4 μM) did not influence the intracellular growth of *L. pneumophila *within alveolar epithelial cells, but affected DNA fragmentation and caspase activation of infected A549 cells.

**Conclusion:**

Infection of A549 alveolar epithelial cells with *L. pneumophila *caused programmed cell death, activation of various caspases, and release of HMGB1. The dot/icm system, a major virulence factor of *L. pneumophila*, is involved in the effects we measured in alveolar epithelial cells. Methyl prednisolone may modulate the interaction of *Legionella *and these cells.

## Background

The Legionnaires' disease bacterium, *Legionella pneumophila*, is one of the most common etiologic agents of bacterial pneumonia. This Gram-negative bacterium can multiply within mononuclear cells *in vivo *and *in vitro *[1], and evade phagosome-lysosome fusion within these cells [2]. An important set of virulence factors expressed by *L. pneumophila *is the dot/icm system, a type IV secretion system that allows the organism to escape phagosome-lysosome fusion and to grow within the phagolysosome [3,4]. The ability of *L. pneumophila *to cause pneumonia is dependent on its capacity to invade and replicate within alveolar macrophages and monocytes [5]. In addition, intracellular replication within alveolar epithelial cells may contribute to the pathogenesis of Legionnaires' disease [5,6].

*Legionella *pneumonia is a potentially serious and life-threatening pneumonia [7,8]. It can exacerbate and develop lethal complications, including acute lung injury (ALI) and acute respiratory distress syndrome (ARDS), a severe form of ALI [9,10]. ARDS is characterized by flooded alveolar air spaces and increased microvascular and epithelial permeability due to neutrophil inflammation, damage to the alveolar capillary endothelium, and disruption of the alveolar epithelium [11,12]. Apoptotic epithelial cells are found in the damaged alveolar epithelium of patients with ARDS [13], implicating such a mechanism in the pathogenesis of ALI and ARDS, including immune recovery and tissue repair after injury [12]. Apoptosis was also induced in *L. pneumophila*-infected alveolar epithelial cells and, consequently, *L. pneumophila *is considered to play a key role in cytotoxicity [14]. However, the apoptotic mechanisms operating in alveolar epithelial cells remain largely unexplored.

This study confirmed the intracellular growth and cytotoxicity of *L. pneumophila *in A549 alveolar epithelial cells. We also investigated the mechanisms of apoptosis of *L. pneumophila*-infected A549 cells, including nuclear deoxyribonucleic acid (DNA) fragmentation and activation of various caspases. In addition, we examined the release of the high-mobility group box 1 (HMGB1) protein, a late phase mediator of acute lung inflammation [15], from *Legionella*-infected alveolar epithelial cells. We also used the avirulent *dotO *mutant strain of *L. pneumophila *lacking a functional dot/icm secretion system [5] to identify bacterial trigger factor(s) for cytotoxicity. Finally, we examined the influence of methyl prednisolone, as an inhibitor of cell injury, on DNA fragmentation, caspase activation, and secretion of HMGB1 from *L. pneumophila*-infected A549 cells.

## Methods

### Bacterial strains

The virulent AA100jm strain of *L. pneumophila *and its avirulent *dotO *mutant have been described previously [5]. The *dotO *mutation severely impairs intracellular growth and evasion of the endocytic pathway by the bacterium [6]. Both *L. pneumophila *strains were grown on buffered charcoal yeast-extract agar medium supplemented with α-ketoglutarate (BCYE-α) at 35°C in a humidified incubator, and subsequently subcultured in buffered yeast extract broth supplemented with α-ketoglutarate (BYE-α).

### Cell culture

The human alveolar epithelial cell line A549 was maintained in RPMI 1640 medium (Nipro, Osaka, Japan) containing 10% heat-inactivated fetal bovine serum (Biological Industries, Kibbutz Beit Haemek, Israel), at 37°C in humidified air under 5% CO_2_.

### Colony assay

Cultured A549 cells in 24-well plates containing 1.25 × 10^5 ^cells/well were infected with *L. pneumophila *at a multiplicity of infection (MOI) of 100. The plates were spun down at 1,300 revolutions per minute (rpm) (about 150 × *g*) for 10 minutes. After incubation for 2 hours, the extracellular fluid and bacteria were removed by washing 3 times with tissue culture medium, and the plates were further incubated for up to 3 days. At various times, the cultured cells were desquamated into the supernatant by gentle scratching with a pipette tip. The supernatant was finally harvested and diluted appropriately with sterile distilled water, and subsequently cultured on BCYE-α agar.

### Cytotoxicity assay

A549 cells were infected with *L. pneumophila *as described for the colony assay, except for MOIs and incubation times. At various times after incubation, the culture supernatants were harvested. Lactate dehydrogenase (LDH) levels were measured in the supernatants as a marker of cytotoxicity using the LDH-cytotoxic Test Wako (Wako Pure Chemical Industries, Osaka, Japan), according to the instructions provided by the manufacturer. The level of specific cytotoxicity was calculated by the following formula:

% of specific LDH release = ([experimental LDH release - the mean of negative control release]/[the mean of positive control release - the mean of negative control release]) × 100.

LDH release from cells treated with 0.05% saponin was used as a positive control, while the negative control was LDH release from nontreated cells.

### Quantitation of high mobility group box 1 (HMGB1) protein

A549 cells were infected with *L. pneumophila *as described for the colony assay. Two days after infection, the culture supernatants were harvested. HMGB1 levels in the supernatants were determined using a sandwich ELISA kit II (Shino-Test Corporation, Kanagawa, Japan) [16] using pig HMGB1 as a standard, according to the instructions provided by the manufacturer. The detection limit was 1 ng/ml.

### TUNEL method

The terminal deoxyribonucleotidyl transferase-mediated triphosphate (dUTP)-biotin nick end labeling (TUNEL) method [17] was used for detection of DNA fragmentation of nuclei. A549 cells grown on glass coverslips in 24-well plates containing 1.25 × 10^5 ^cells/well were infected with *L. pneumophila *at various MOIs. Positive controls were treated with 30 μM mitomycin C. After incubation for 2 days, the glass coverslips were harvested, fixed with 4% paraformaldehyde, and washed with PBS. The cells were permeabilized with 0.5% Tween 20 and treated with MEBSTAIN Apoptosis Kit Direct (Medical and Biological Laboratories Co, Nagoya, Japan). Cells were then treated with RNase and propidium iodide (PI). The nick end labeling was analyzed with a confocal laser scanning microscope (Fluorview, Olympus, Tokyo). TUNEL positive cells were also quantitated using flow cytometry (Flow Cytometer, Coulter Corporation, FL).

### Colorimetric assay for caspase activity

Commercially available caspase activity assays (Colorimetric Assay kit; BioVision Research Products, Mountain View, CA) based on colorimetric detection of cleaved para-nitroaniline-labeled substrates specific for caspase 3 (DEVD), 8 (IETD), 9 (LEHD), and 1 (YVAD) were used to analyze caspases activity according to the instructions provided by the manufacturer. Briefly, A549 cells cultured in 8.5-cm dishes containing 7 × 10^6^/dish were stimulated, collected, and lysed on ice. Cleaved samples (4 μg/μL), were incubated at 37°C for 2 hours in the presence of labeled caspase-specific substrate conjugates for caspase 3, 8, 9, and 1. Caspase activity was determined from the sample absorbance at 405 nm measured in a microplate reader (Bio-Rad, Tokyo, Japan).

### Western blotting

A549 cells were infected with *L. pneumophila *or treated with mitomycin C, in a manner similar to caspase activity analysis. After 24 hours, the cells were lysed in a buffer containing 62.5 mM Tris-HCl (pH 6.8), 2% sodium dodecyl sulfate, 10% glycerol, 6% 2-mercaptoethanol, and 0.01% bromophenol blue. Equal amounts of protein (20 μg) were subjected to electrophoresis on sodium dodecyl sulfate-polyacrylamide gels followed by transfer to a polyvinylidene difluoride membrane and probing sequentially with specific antibodies against caspases 8 and 9, and against cleaved poly (ADP-ribose) polymerase (PARP), which is a natural substrate of caspase 3 (Cell Signaling Technology Inc, Danvers, MA). The bands were visualized by enhanced chemiluminescence (Amersham Biosciences, Piscataway, NJ).

### Influence of methyl prednisolone on DNA fragmentation, caspase activity, and HMGB1 release

A549 cells were pretreated with or without methyl prednisolone (53.4 μM) for one day, and subsequently stimulated. Cells were then further incubated with or without methyl prednisolone (53.4 μM). DNA fragmentation, caspase activity, and HMGB1 release were assayed in *L. pneumophila*-infected A549 cells following these treatments. DNA fragmentation was analyzed by flow cytometry, while caspase activity and HMGB1 release were determined as described above.

### Statistical analysis

Statistical significances were determined using the unpaired or paired t-test (for two-category comparison), or ANOVA and SNK test as post hoc test (for comparison of more than three parameters). A significant difference was considered to be *P *< 0.05.

## Results

### Intracellular growth and cytotoxicity of *L. pneumophila *in A549 cells

**Figure 1:**
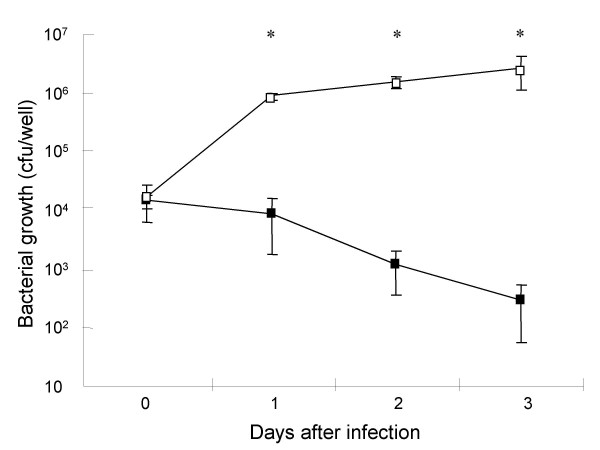
**Intracellular growth of *L. pneumophila *in A549 cells**. A549 cells were infected with *L. pneumophila *virulent AA100jm and avirulent *dotO *mutant strains at an MOI of 100. After 2 hours incubation, extracellular bacteria were removed by washing, and the infected cells were cultured further. The number of viable bacteria in each well was determined by the CFU counting method. Symbols : ▫, virulent strain AA100jm; ▪, avirulent strain *dotO *mutant. Data are mean ± SD of four wells. * *P *< 0.05.

First, we verified the infection and intracellular growth of *L. pneumophila *in A549 cells. Intracellular growth of the virulent strain was observed 1 day after infection, subsequently increasing to approximately 100-fold the initial growth 3 days after infection. In contrast, the avirulent *dotO *mutants did not multiply intracellularly and their growth decreased with time. Cells infected with the two different strains therefore showed significantly different viable bacterial burdens from one day after infection (Fig. 1).

**Figure 2:**
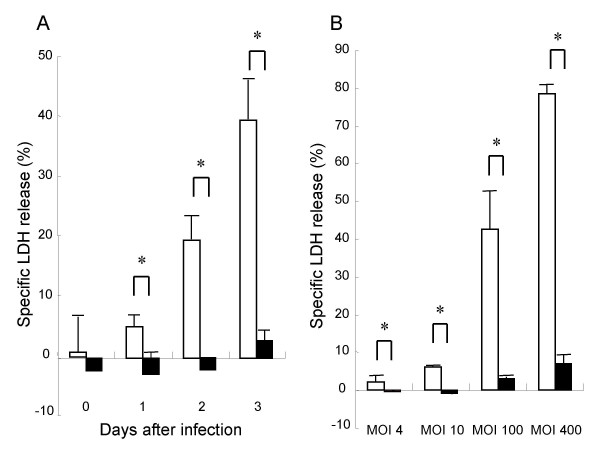
**Cytotoxic effect of *L. pneumophila *on A549 cells**. A549 cells were infected with *L. pneumophila *virulent AA100jm and avirulent *dotO *mutant strains. LDH levels in the cell supernatants showed a time-dependent change after infection with *L. pneumophila *at an MOI of 20 (A), and an MOI dose-response relationship 2 days after infection (B). Symbols : ▫, virulent strain AA100jm; ▪, avirulent strain *dotO *mutant. Data are mean ± SD of three wells. * *P *< 0.05.

**Figure 3:**
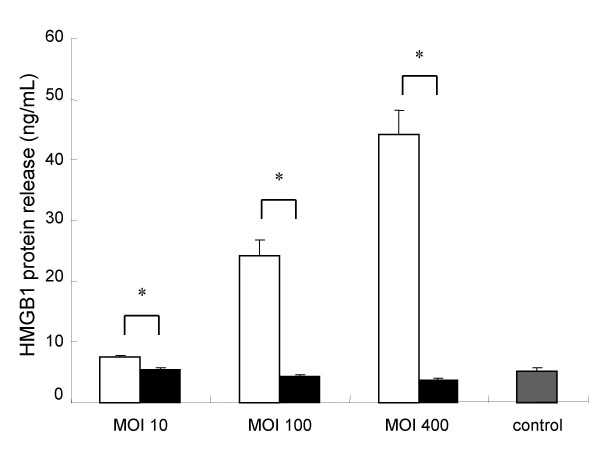
**HMGB1 release from A549 cells induced by *L. pneumophila *infection**. A549 cells were infected with *L. pneumophila *virulent AA100jm and avirulent *dotO *mutant strains. After incubation, HMGB1 levels in the supernatant showed an MOI dose-response relationship 2 days after infection. Symbols : ▫, virulent strain AA100jm; ▪, avirulent strain *dotO *mutant. Data are mean ± SD of three wells. * *P *< 0.05.

To determine the cytotoxic effect of *L. pneumophila *in A549 cells, we measured LDH level in the supernatants. As for LDH levels, time-dependent and MOI dose-dependent increases of cytotoxicity in the AA100jm-infected A549 cells were significantly observed, compared to those in the *dotO *mutant-infected cells (Fig. 2a and 2b). A newly defined cytokine, HMGB1 is reported to be released during cell death. MOI dose-dependent significant increases of HMGB1 concentration were observed in A549 cells infected with virulent strain, compared to those in cells infected with the avirulent strain (Fig. 3).

### Nuclear DNA fragmentation of *L. pneumophila*-infected A549 cells

**Figure 4:**
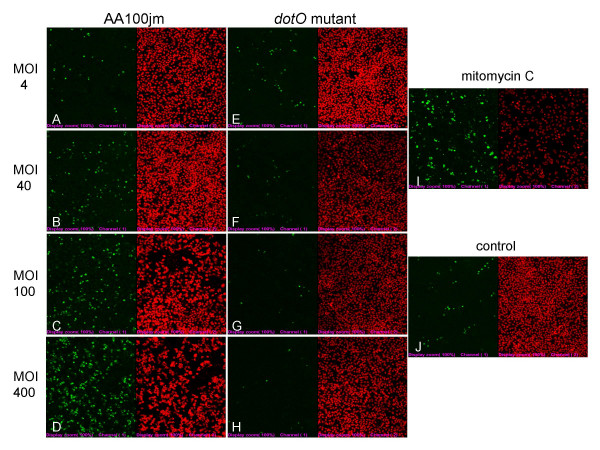
**Nuclear DNA fragmentation of *L. pneumophila*-infected A549 cells detected by the TUNEL method**. MOI dose-response relationship of nuclear DNA fragmentations 2 days after infection with the virulent AA100jm strain (A-D) and the avirulent *dotO *mutant strain (E-H). Nuclear DNA fragmentation of cells 2 days after exposure to 30 μM mitomycin C (I) and with no treatment (control) (J). Cells were observed with a confocal laser scanning microscopy (all 200 ×). The nuclear DNA fragmentation is shown in green (FITC staining), and A549 cell nuclei in red (PI staining).

**Figure 5:**
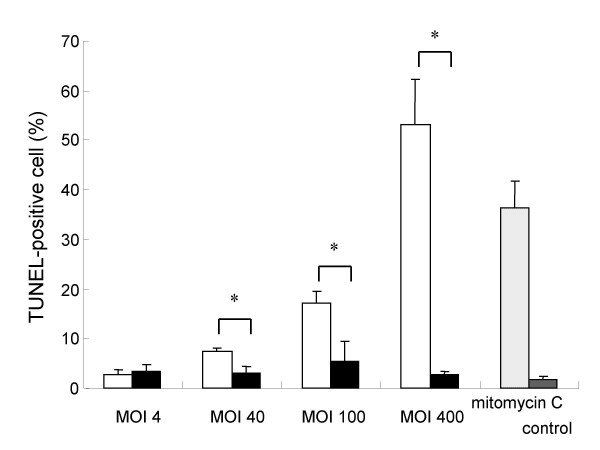
**Nuclear DNA fragmentation of *L. pneumophila*-infected A549 cells detected by the TUNEL method**. TUNEL-positive A549 cell numbers with each stimulus are presented. Cells were observed with a confocal laser scanning microscopy. More than 500 cells were counted from 10 randomized high-power fields, and TUNEL-positive cells were expressed as a ratio per total number of cells. Symbols: ▫, virulent strain AA100jm; ▪, avirulent strain *dotO *mutant. Data are mean ± SD of three different experiments. * *P *< 0.05.

**Figure 6:**
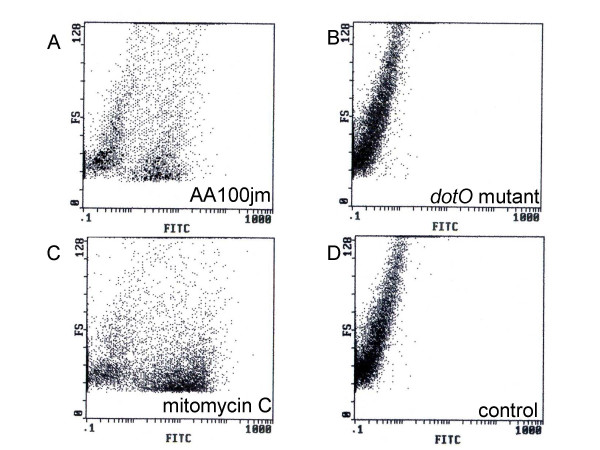
**Analysis of the nuclear DNA fragmentation in *L. pneumophila*-infected A549 cells by flow cytometry**. Two days after treatment, A549 cells were treated for TUNEL staining and the nuclear DNA fragmentation was analyzed by flow cytometry. A549 cells infected with the virulent AA100jm (A) and the avirulent *dotO *mutant (B) strains at an MOI of 100, exposed to 30 μM mitomycin C (C), or not stimulated (control) (D) are shown. The nuclear DNA fragmentation is expressed as FITC staining intensity. FS indicates the cell sizes.

We investigated nuclear DNA fragmentation in infected A549 cells as a possible mechanism of *L. pneumophila*-induced cytotoxicity by TUNEL staining. A549 cells infected with the virulent strain of *L. pneumophila*, AA100jm, showed significantly more nuclear DNA fragmentation than those carrying the avirulent *dotO *mutant strain (Fig. 4a–h and Fig. 5). The fragmentation also increased dose-dependently with MOI in the AA100jm-infected cells (Fig. 4a–d and Fig. 5). Furthermore, these results were replicated in flow-cytometry analyses of infected A549 cells (Fig. 6a and 6b).

### Caspase activity in *L. pneumophila*-infected A549 cells

**Figure 7:**
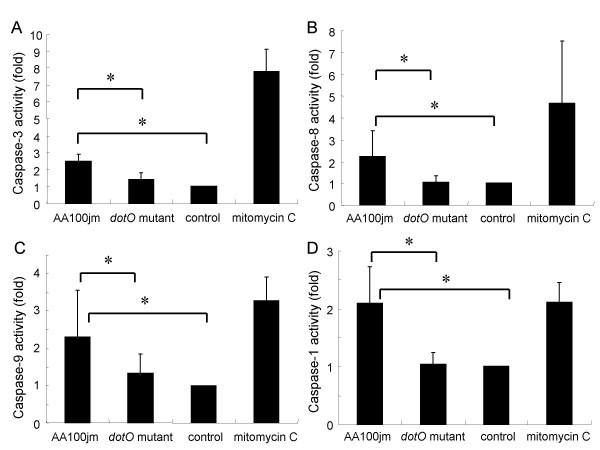
**Caspase activity in *L. pneumophila*-infected A549 cells detected by colorimetric assay**. One day after stimulation, infection with the virulent strain AA100jm and the avirulent strain *dotO *mutant at an MOI of 400, expose to 30 μM mitomycin C and no-stimulation (control), caspases activity of each cell-group was detected by colorimetric assay. The activities of caspase 3 (A), 8 (B), 9 (C), and 1 (D) are shown. Data are mean ± SD of five or six different experiments. * *P *< 0.05.

**Figure 8:**
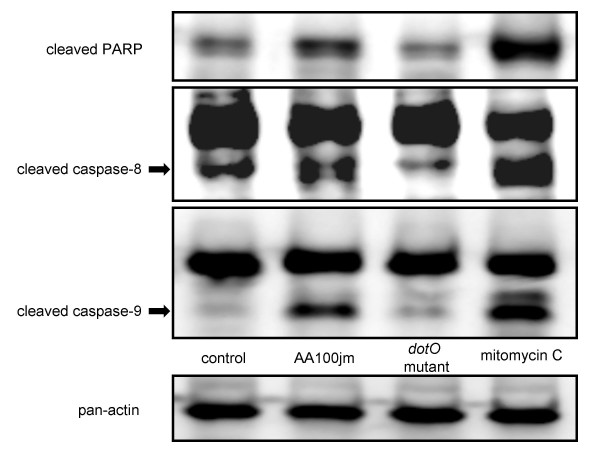
**Caspase activity in *L. pneumophila*-infected A549 cells detected by western blotting**. Cells treated as for the colorimetric assay were subjected to western blotting. Cleavage activations of caspase 8, 9, and poly (ADP-ribose) polymerase (PARP; natural substrate of caspase 3) were detected.

Caspase activation is essential for DNA fragmentation in apoptosis induced by a variety of stimuli [18,19]. We therefore measured the activity of various caspases in *L. pneumophila*-infected A549 cells colorimetrically. A549 cells infected with the virulent AA100jm strain had significantly elevated caspase 3, 8, 9, and 1 activities compared to those infected with the *dotO *mutant bacteria (Fig. 7a–d). To further demonstrate caspase activity in *L. pneumophila*-infected A549 cells, we examined the cleavage activation of various caspases by western blot analysis. These experiments confirmed cleaved products of caspase 8, and 9, and the natural substrate of caspase 3, PARP, in cells infected with the virulent strain of *L. pneumophila *(Fig. 8).

### Inhibition of nuclear DNA fragmentation, caspase activation, and HMGB1 release in A549 cells by methyl prednisolone

**Figure 9:**
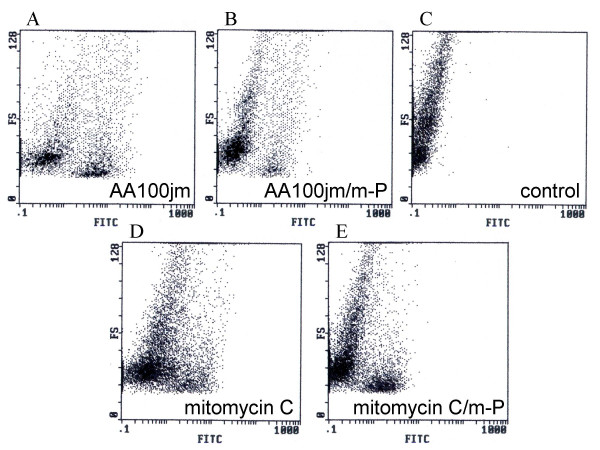
**Influence of methyl prednisolone on the nuclear DNA fragmentation of *L. pneumophila*-infected A549 cells**. Cells were pre-treated with and without methyl prednisolone (53.4 μM) for one day, and subsequently stimulated. After a 2-day incubation, nuclear DNA fragmentation stained by the TUNEL method was analyzed by flow cytometry. The nuclear DNA fragmentation of virulent strain AA100jm-infected cells without (A) and with methyl prednisolone (B), is shown with no-pretreatment/no-stimulation cells (control) (C), and 30 μM mitomycin C-exposed cells without (D) and with methyl prednisolone (E). FS indicates the cell sizes. m-P; methyl prednisolone.

**Figure 10:**
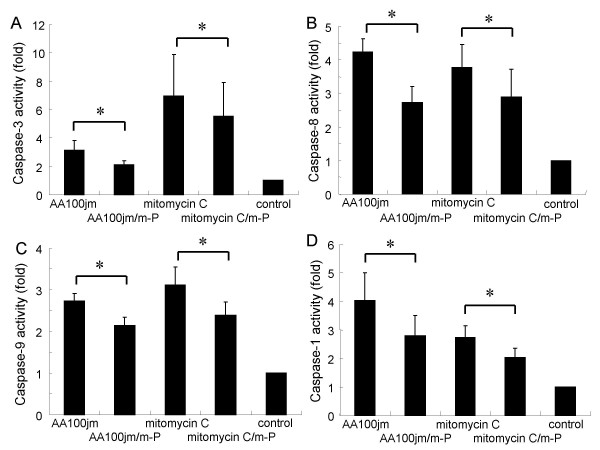
**Influence of methyl prednisolone on caspase activity in *L. pneumophila*-infected A549 cells**. Cells were pre-treated with and without methyl prednisolone (53.4 μM) for one day, and subsequently stimulated. After a 1-day incubation, caspases activity was measured colorimetrically. The activities of caspase 3 (A), 8 (B), 9 (C), and 1 (D) are presented. Data are mean ± SD of four or six different experiments. * *P *< 0.05. m-P; methyl prednisolone.

**Figure 11:**
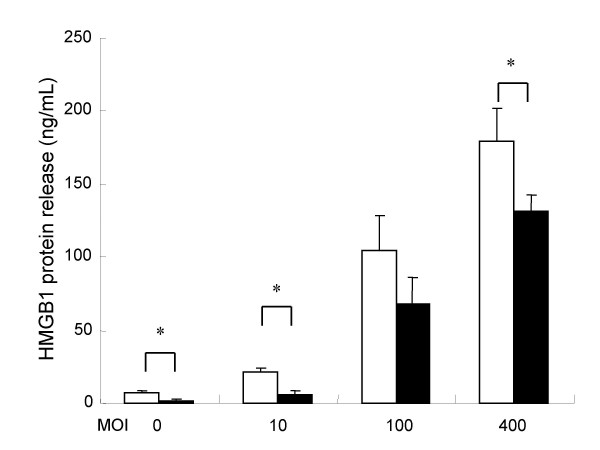
**Influence of methyl prednisolone on HMGB1 release from *L. pneumophila*-infected A549 cells**. Cells were pre-treated with and without methyl prednisolone (53.4 μM) for one day, and subsequently infected with the virulent AA100jm strain of *L. pneumophila*. After another day, HMGB1 release was measured in cell supernatants. Symbols: ▫, without methyl prednisolone; ▪, with methyl prednisolone. Data are mean ± SD of three different experiments. * *P *< 0.05.

Glucocorticoid-induced antiapoptotic signaling was recently associated with resistance to apoptosis in cells of epithelial origin [20]. We found that methyl prednisolone partly inhibited DNA fragmentation (Fig. 9a and 9b), as well as significantly reducing caspase 3, 8, 9, and 1 activities (Fig. 10a–d) and HMGB1 release (Fig. 11) in AA100jm-infected cells.

## Discussion

Cell death is typically discussed as necrosis, apoptosis, or pyroptosis. While necrosis is characterized as accidental cell death due to physical damage, apoptosis is a strictly regulated genetic and biochemical suicide program that is critical during development and tissue homeostasis, and in modulating the pathogenesis of a variety of diseases [21]. A number of pathogens cause host cell death with features of apoptosis [22-24]. Pyroptosis is a recently described type of cell death, in which caspase 1 is activated and inflammatory cytokines are released as cells are dying [25].

Several previous studies investigated the induction of apoptosis correlated with cytotoxicity in *Legionella*-infected cells [26,27,14]. The present study confirmed that *L. pneumophila *multiply within A549 alveolar epithelial cells, resulting in cytotoxicity. We investigated the mechanism by which *L. pneumophila *induces cell injury in A549 cells by the TUNEL method using both confocal laser scanning microscopy and flow cytometric analysis to assess DNA fragmentation at the single cell level. We also assayed caspase activation using colorimetric assays and western blotting. Chromosomal DNA fragmentation that increased dose-dependently with MOI, and the activation of caspase 3, 8, and 9, indicated that some alveolar epithelial cell injury induced by *L. pneumophila *was attributable to apoptosis. The results suggested that activation of caspase 1 within alveolar epithelial cells might also be involved.

The *L. pneumophila *mutant strain carrying a defective dot/icm system failed to induce chromosomal DNA fragmentation or caspase activation in A549 cells. However, further studies are needed to ascertain whether the induction of apoptosis in these cells following *L. pneumophila *infection is dependent on the dot/icm system itself or on the intracellular growth capacity of the bacteria. Gross et al. [28] suggested that certain intracellular bacteria might inhibit apoptosis to enhance their own survival, thus boosting replication within phagocytes and their continued presence at sites of infection. Another group of bacteria including *L. pneumophila *promotes the apoptosis of phagocytes, resulting in either control of intracellular growth of the bacteria or evasion of the immune system [14]. Based on the results of the present study, it is difficult to tell whether caspase activation in alveolar epithelial cells works to regulate or augment the infection. Further study is clearly warranted to pursue this proposition.

The caspase family of cysteine proteases is important in regulating apoptosis and the inflammatory response [29]. Caspase 3, main executioner caspase, is specifically required for DNA fragmentation leading to the typical apoptotic pattern of DNA laddering [30-32]. Otherwise, initiator caspases appear to be activated by many apoptosis-inducing stimuli via two major pathways: the death receptor pathway and the mitochondrial/apoptosome pathway [33]. The regulatory protein caspase 8 is directly activated by death receptors, while caspase 9 activation follows mitochondrial stress [34,35]. Our results here demonstrated for the first time that *L. pneumophila *induced caspase-dependent cell injury in A549 cells with elevations in caspase 3, 8, and 9 activities. Gao and Abu [36] demonstrated that the induction of apoptosis by *L. pneumophila *in macrophages is mediated through activation of caspase 3, while Fischer et al. [37] similarly implicated caspase 9 and 3 in myeloid cells and T cells.

Some caspases, such as caspase 1, are also important components of signaling pathways associated with the immune response to microbial pathogens. Caspase 1 activation is associated with the maturation of pro-inflammatory cytokines, such as interleukin-1β (IL-1β) and IL-18, but not apoptosis *per se *[38]. Recent studies on *Shigella *and *Salmonella *infections implicated caspase 1 activation in programmed cell death [38,39] that was different from apoptosis induced by the activation of caspase 3 [40]; this process was named pyroptosis [25]. Our present study found that caspase 1 was activated by *L. pneumophila *infection in A549 alveolar epithelial cells, suggesting a correlation with the cytopathic effect on the cells.

This is the first description also of increased HMGB1 protein in the supernatants of alveolar epithelial cells infected with virulent *L. pneumophila*. HMGB1 is a non-histone nuclear protein with dual function. Inside the cell, HMGB1 binds DNA and regulates transcription, whereas it acts as a cytokine outside the cell [41,42]. HMGB1 leaks out from necrotic cells and signals to neighboring cells that tissue damage has occurred [43], and recent reports indicate that HMGB1 might also be released during apoptosis [44]. Therefore, HMGB1 protein is a cytokine released from dying cells, but it is not clear which type(s) of cell death is associated with release of HMGB1 from *Legionella*-infected alveolar epithelium. In this study, the increased LDH and HMGB1 secreted from AA100jm-infected A549 cells was dependent on the MOI and correlated with an increase in TUNEL-positive cells. This finding suggests a link between HMGB1 release from dying cells and caspase activation.

Glucocorticoids have a dual effect on apoptosis. Cells of hematopoietic origin such as monocytes, macrophages, lymphocytes, and lymphoma cells are very sensitive to glucocorticoid stimulation of apoptosis [20]. In addition, recent studies associated glucocorticoid-induced anti-apoptotic signaling with apoptosis resistance in transformed cells of epithelial origin [20,45]. We also confirmed that chromosomal DNA fragmentation of AA100jm-infected A549 cells is inhibited by methyl prednisolone, and that this inhibition of cell injury is accompanied by the degradation of caspase 3, 8, and 9. We therefore postulate that the inhibition of caspase-dependent cell injury by methyl prednisolone resulted at least partly from the depressed death receptor and mitochondrial signaling. Moreover, we detected inhibition of caspase 1 in our experiments, which probably related to signaling pathways associated with immune responses to microbial pathogens.

The present *in vitro *study showed potential pathogenesis of *L. pneumophila *against human alveolar epithelial cells. *L. pneumophila *clearly induced the damage of alveolar epithelial cells in dose-dependent and time-dependent manners. The administration of methyl prednisolone at the early stage of *L. pneumophila *infection may decrease apoptosis in alveolar epithelial cells. Clinical significance of alveolar epithelial cells infection has been pointed out with other *Legionella *spp., such as *Legionella dumoffii *[46], but its role in *L. pneumophila *infection is not so clear. The present findings warrant further *in vivo *animal studies and human studies.

## Conclusion

Infection of A549 alveolar epithelial cells by *L. pneumophila *caused cell death, nuclear DNA fragmentation, activation of various caspases, and release of HMGB1. The dot/icm system was identified as a major virulence factor for the effects of *L. pneumophila *on these cells. This study suggested that the cytopathic effect of *L. pneumophila *on A549 alveolar epithelial cells is mediated via activation of caspase 3, 8, 9, and 1. Therefore, the mode of cell death could be apoptosis and/or pyroptosis, induced by either death-receptor signaling or mitochondrial stress. In this study, methyl prednisolone had an anti-apoptotic effect on alveolar epithelial cells infected with bacteria. The search for substances that modulate the interaction between alveolar epithelial cells and *Legionella *may be warranted as a novel therapeutic intervention.

## Competing interests

The authors declare that they have no competing interests.

## Authors' contributions

MF carried out all experiments and was involved in the design and coordination of the study and drafting the manuscript. FH measured HMGB1 levels, and was involved in the design and coordination of the study and drafting the manuscript. KH, MA, SH, SY, MK and MT were involved in the design and coordination of the study. HT and NM were involved in western blot analyses. JF was involved in the design and coordination of the study and drafting the manuscript. All authors read and approved the final manuscript.
